# LINC01605 Predicts the Poor Prognosis of Non‐Small Cell Lung Cancer and Promotes Chemotherapy Resistance by Regulating miR‐7111‐5p/ELK1

**DOI:** 10.1002/kjm2.70224

**Published:** 2026-05-15

**Authors:** Fang Zhang, Zhi‐Liang Yang, Tian‐Tian Qin, Guo‐Jun Zhang, Xiang Li

**Affiliations:** ^1^ Department of Oncology The Fourth Hospital of Changsha (Integrated Traditional Chinese and Western Medicine Hospital of Changsha, Changsha Hospital of Hunan Normal University) Changsha China; ^2^ Department of Cardiothoracic Surgery People's Hospital of Kaizhou District Chongqing China; ^3^ Department of Pulmonary and Critical Care Medicine The First Affiliated Hospital of Zhengzhou University Zhengzhou China; ^4^ Department of Pulmonary and Critical Care Medicine Zhengzhou Central Hospital Affiliated to Zhengzhou University Zhengzhou China; ^5^ Department of Respiratory and Critical Care Medicine Health Community Group of Yuhuan Second People's Hospital Taizhou China

**Keywords:** chemotherapy resistance, ELK1, LINC01605, miR‐7111‐5p, non‐small cell lung cancer

## Abstract

Non‐small cell lung cancer (NSCLC) is characterized by high incidence, mortality, and poor patient prognosis, with chemotherapy resistance being a common challenge. This research intends to identify key molecules involved in NSCLC and elucidate the regulatory role of the LINC01605/miR‐7111‐5p/ELK1 axis in chemotherapy resistance. LINC01605 and its downstream targets were predicted using the lncRNASNP2‐human and miRDB databases. RT‐qPCR was employed to measure the expression of LINC01605 across different NSCLC patients, and its prognostic value was assessed through ROC curve, Kaplan–Meier curve, and Cox regression. Dual‐luciferase reporter assay was conducted to verify interactions between miR‐7111‐5p and LINC01605 or ELK1. Transwell assay evaluated cell invasion capabilities, while CCK8 assay confirmed changes in chemotherapy drug sensitivity in chemotherapy‐resistant cells across different treatment groups. Elevated expression of LINC01605 was observed in NSCLC patients, correlating with reduced survival rates, and its expression was further elevated in patients with chemotherapy resistance. The upregulation of miR‐7111‐5p inhibited the proliferation and invasion of NSCLC cells induced by LINC01605. Mechanistically, LINC01605 negatively regulated miR‐7111‐5p expression while positively influencing ELK1 expression. Silencing ELK1 and upregulating miR‐7111‐5p levels reversed the chemotherapy resistance of A549 cells induced by LINC01605. By targeting the miR‐7111‐5p/ELK1 regulatory axis, LINC01605 induced chemotherapy resistance in NSCLC, highlighting its potential as a significant biomarker for NSCLC.

## 1 Introduction

Lung cancer stands as one of the most deadly malignancies worldwide, with approximately 2 million new cases and 1.76 million deaths annually [1]. Histologically, it is mainly divided into small cell lung cancer (SCLC) and NSCLC, with the latter accounting for approximately 85% of all cases [2]. The pathogenesis of NSCLC is multifaceted, involving long‐term smoking, air pollution, occupational exposure, genetic predisposition, and viral infections [3]. These intertwined risk factors contribute to its high incidence and complexity. NSCLC often presents insidiously and is typically diagnosed at advanced stages, leading to a poor prognosis. Only 25% of NSCLC patients survive beyond 5 years after diagnosis, with stage IV patients having a mere 4%–6% five‐year survival rate [4, 5]. Thus, the development of effective therapeutic strategies for NSCLC is a pressing global public health challenge.

Chemotherapy remains a cornerstone treatment for patients with advanced NSCLC, with Cisplatin (DDP) and paclitaxel (PTX) being commonly used chemotherapeutic agents for NSCLC [6]. In recent years, multiple kinase inhibitor targets have been identified and applied clinically, including epidermal growth factor receptor (EGFR), anaplastic lymphoma kinase (ALK), and ROS proto‐oncogene 1 (ROS1). EGFR mutations are particularly prevalent in NSCLC [7]. Epidermal growth factor receptor tyrosine kinase inhibitors (EGFR‐TKIs) are employed to treat NSCLC patients with EGFR mutations. The third‐generation EGFR inhibitor osimertinib (Osi) has demonstrated clinically significant improvements; yet, like other targeted therapies, it inevitably faces resistance development [8, 9]. Most patients develop secondary mutations after a period of TKI treatment, leading to drug resistance. Thus, overcoming drug resistance and optimizing treatment strategies are urgent challenges.

In recent years, increasing evidence has underscored the potential of lncRNAs as cancer biomarkers [10]. For example, SNHG14 is highly expressed in NSCLC and promotes cell proliferation, invasion, and migration [11]. LncRNA BC200 regulates cisplatin resistance in NSCLC through the PI3K/AKT pathway [12]. Previous studies have demonstrated that LINC01605 modulates breast cancer by targeting LDHA to mediate aerobic glycolysis [13], and promotes malignant progression in laryngeal squamous cell carcinoma by targeting miR‐493‐3p [12]. Given its established role in cancer development, we investigated the function of LINC01605 in NSCLC, a previously unexplored area. We also investigated the role of LINC01605 in chemoresistance of NSCLC through the lncRNA‐miRNA‐mRNA axis and explored the underlying regulatory mechanism of LINC01605 in chemoresistance. These findings offer insights and strategies for combating chemoresistance and treating NSCLC.

## 2 Materials and Methods

### 2.1 Clinical Samples

All serum samples were collected from The First Affiliated Hospital of Zhengzhou University. This study included a total of 270 NSCLC patients. Meanwhile, 270 healthy volunteers and 270 patients with pneumonia who were matched in terms of gender, age, and smoking history were also included as controls. Based on the criteria for evaluating the efficacy of solid tumors, NSCLC patients were categorized into the chemotherapy‐sensitive group and the chemotherapy‐resistant group. Complete response (CR) and partial response (PR) were regarded as chemotherapy‐sensitive. Stable disease (SD) and progressive disease (PD) were considered chemotherapy‐resistant. The study was performed in line with the principles of the Declaration of Helsinki. Approval was granted by the Ethics Committee of The First Affiliated Hospital of Zhengzhou University before the study began. Written informed consent was obtained from all participants.

### 2.2 Predicting Potential Targets

The dataset GSE262582 about Osi resistance was screened from the GEO database (https://www.ncbi.nlm.nih.gov/geo/), and LINC01605 with the largest log2FC was selected as the research object. The downstream miRNAs were predicted by the lncRNASNP2‐human database (https://guolab.wchscu.cn/lncRNASNP#!/), and miR‐7111‐5p, with the lowest binding energy, was chosen as the downstream of LINC01605. The miRDB database (https://mirdb.org/custom.html) was then employed to predict downstream targets of miR‐7111‐5p, with genes scoring ≥ 90 considered as potential targets. KEGG enrichment analysis revealed that these genes were involved in multiple pathways, with ELK1 showing a strong association with human cancers.

### 2.3 RT‐qPCR

Total RNA was extracted using either the miRNeasy Serum/Plasma Kit (Qiagen, Japan) or TRIzol Reagent (Invitrogen, USA). Reverse transcription was performed using HiScript III RT SuperMix for qPCR or the miRNA 1st Strand cDNA Synthesis Kit (Vazyme, Nanjing, China), following the manufacturer's instructions. RT‐qPCR amplification was carried out using SYBR Premix Ex Taq II (Takara, Japan), and data were normalized and quantified using the 2^−ΔΔCt^ method, with GAPDH and U6 serving as internal controls. The primer sequences are as follows: LINC01605: F: TACAAACAGCCGACCTTCCT, R: ACAAGACCACACATGGCTAGG; ELK1: F: TCCCTGCTTCCTACGCATACA, R: GCTGCCACTGGATGGAAACT; GAPDH: F: GAGAAGGCTGGGGCTCATTT, R: AGTGATGGCATGGACTGTGG; miR‐7111‐5p: F: 5′‐AGGACAGGCCATCTG‐3′; U6: F: 5′‐CTCGCTTCGGCAGCACA‐3′, R: 5′‐AACGCTTCACGAATTTGCGT‐3′.

### 2.4 Cell Culture and Transfection

BEAS‐2B cells were cultured in specific media (Beyotime, Shanghai, China). To establish Osi/A549 cell lines, parental cells in logarithmic growth phase were subjected to chronic, stepwise exposure to increasing concentrations of Osi, ranging from 5 nM to 3000 nM. All other cells were purchased from Procell (Wuhan, China). HCC827 and A549 cells were cultured in RPMI 1640 or DMEM medium (Solarbio, Beijing, China) at 37°C with 5% CO_2_. Specifically, A549/DDP and A549/PTX cells were cultured in Ham's F‐12 K medium (Procell, Wuhan, China), while the HCC827/PTX cell line, obtained from BioVector Preservation Center, was cultured in DMEM medium. Cell transfections, including siLINC01605, siELK1, miR‐7111‐3p mimics and LINC01605pcDNA, were performed using Lipofectamine 2000 (Thermo Fisher, USA).

### 2.5 CCK8

Cells were seeded into 96‐well plates and allowed to adhere. After treatment with drugs at various concentrations, 10 μL of CCK 8 reagent (Biosharp, #BS350B, China) was added to each well, and absorbance was measured after incubation for 2 h.

### 2.6 Transwell

Cells were seeded into chambers coated with 10% Matrigel (BD Biosciences, USA) and placed in the upper chamber of a Transwell plate. Following different treatments, medium containing 15% FBS was added to the lower chamber. After 24 h, invasive cells on the lower surface were fixed, stained with crystal violet, and counted using a Transwell migration assay.

### 2.7 Dual‐Luciferase Reporter Assay

Cells were co‐transfected with 3′‐UTR‐specific luciferase reporter constructs and miR‐7111‐5p mimics using lipofectamine 2000. After 48 h, luciferase activity was measured using the Dual Luciferase Assay kit (Thermo, USA).

### 2.8 RNA Pull‐Down

A549 cells were transfected with a biotin‐labeled LINC01605 fragment, followed by trypsin digestion and homogenization. Cell lysates were incubated with streptavidin‐coated magnetic beads (Sigma‐Aldrich, USA) at 4°C for 2 h to perform RNA pull‐down. Bound LINC01605 or ELK1 mRNA was purified using the RNeasy mini kit (Qiagen, Germany), and miR‐7111‐5p expression in the bound fraction was detected via RT‐qPCR.

### 2.9 Western Blotting

Cell lysates were prepared from transfected or drug‐resistant A549 cells using RIPA lysis buffer (Thermo, USA). The protein solution was obtained by collecting the supernatant after centrifuging (12,000*g*) at 4°C for 20 min. Protein content was quantified using the BCA assay kit (Solarbio), and proteins were separated by SDS‐PAGE. After transfer to PVDF membranes, proteins were blocked and incubated with primary and secondary antibodies. Finally, proteins were detected using the ECL chemiluminescent kit (Beyotime).

### 2.10 Statistical Analysis

All statistical analyses were conducted using GraphPad Prism 9.0 software and SPSS 23.0 software. Data from three independent experiments are presented as mean ± standard deviation (SD), with statistical significance set at *p* < 0.05.

## 3 Results

### 3.1 High Expression of LINC01605 Predicts Chemotherapy Resistance in NSCLC

**FIGURE 1:**
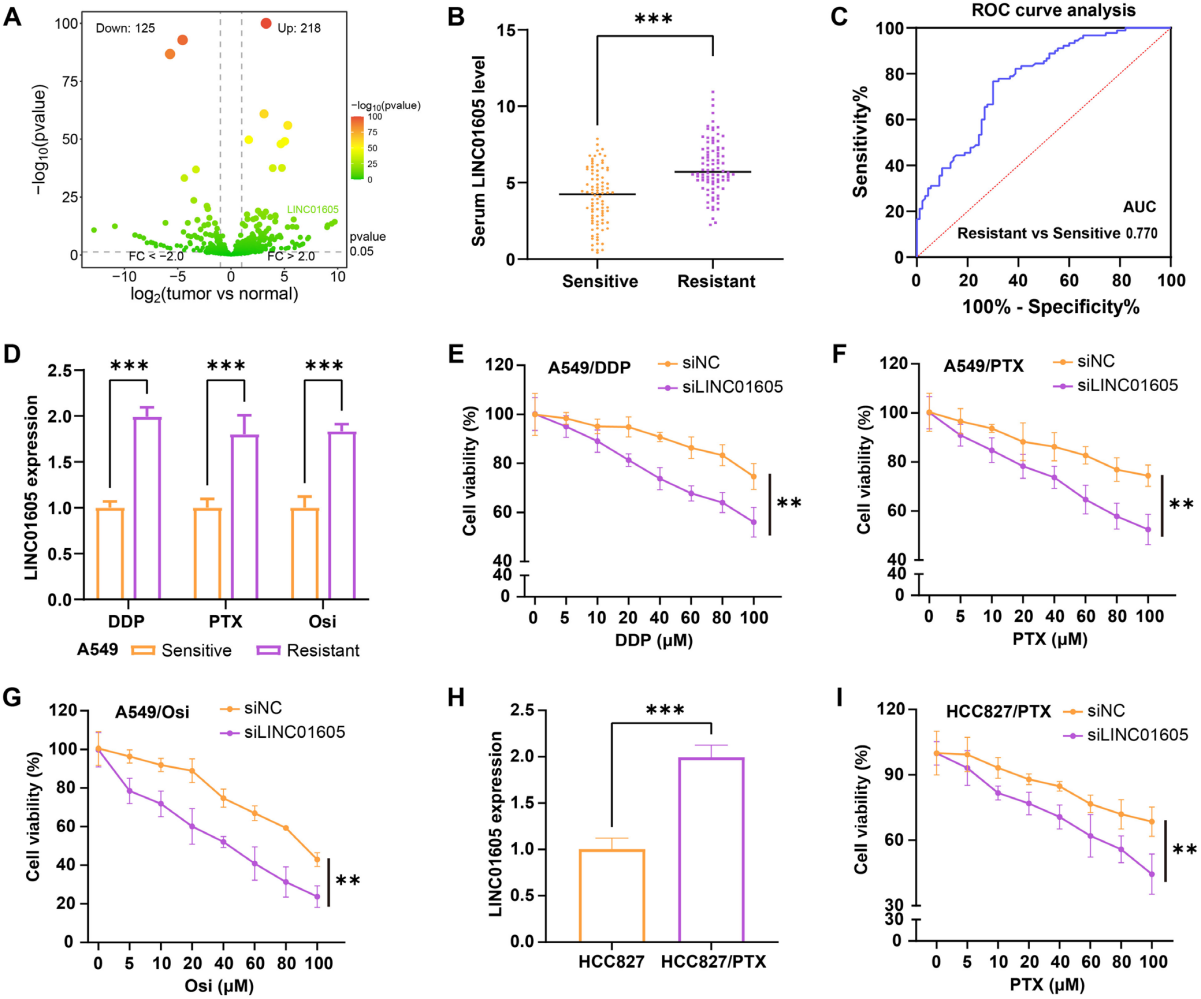
High Expression of LINC01605 Predicts Chemotherapy Resistance in NSCLC. (A) Volcano plot of lncRNA expression in the GSE262582 dataset. (B) Expression of LINC01605 in the patient serum. (C) ROC curve of serum LINC01605 for differentiating patients with chemotherapy sensitivity and resistance. (D) LINC01605 was highly expressed in resistant A549 cells. (E–G) Silencing LINC01605 increased the sensitivity of drug‐resistant cells to chemotherapy drugs. (H) The expression of LINC01605 in PTX‐resistant cells. (I) Silencing LINC01605 increased the sensitivity of HCC827/PTX cells to PTX. **p < 0.01; ***p < 0.001.

A volcano plot of lncRNA expression was generated from the GSE262582 dataset (Figure 1A), identifying LINC01605 as the lncRNA with the highest log2FC. Chi‐square testing revealed a significant association between high LINC01605 expression and EGFR mutations (Table 1), which are key contributors to chemotherapy resistance in NSCLC. This suggests a potential link between LINC01605 and chemoresistance. Subsequently, RT‐qPCR analysis confirmed elevated LINC01605 levels in chemotherapy‐resistant patients (Figure 1B). Using resistant patients as positive cases and sensitive patients as negative controls, an ROC curve was constructed, yielding an AUC of 0.770 (Figure 1C), indicating that LINC01605 is a potential biomarker for chemotherapy resistance in NSCLC.

**TABLE 1:** The relationship between serum LINC01605/miR‐7111‐5p and the clinical characteristics of NSCLC patients.

		Serum LINC01605		*p* *	Serum miR‐7111‐5p		*p* *
		Low (135)	High (135)		Low (135)	High (135)	
T stage	T1/2	69	61	0.330	56	74	0.028
	T3/4	66	74		79	61	
N stage	N0	75	63	0.144	61	77	0.051
	N1/2/3	60	72		74	58	
M stage	M0	70	44	0.001	46	68	0.007
	M1	65	91		89	67	
EGFR mutation	Negative	81	46	< 0.001	54	73	0.021
	Positive	54	89		81	62	
Histological type	LUSC	71	75	0.625	70	76	0.464
	LUAD	64	60		65	59	
Age	< 60	64	72	0.330	72	64	0.330
	≥ 60	71	63		63	71	
Gender	Female	74	63	0.181	65	72	0.394
	Male	61	72		70	63	
Smoking	No	62	65	0.715	68	59	0.272
	Yes	73	70		67	76	
Family history	No	68	63	0.543	72	59	0.113
	Yes	67	72		63	76	

Abbreviations: LUAD = lung adenocarcinoma; LUSC = lung squamous cell carcinoma; M = distant metastasis; N = lymph node metastasis; T, tumor.

* *p* = Chi‐square test.

To further validate LINC01605's role in chemoresistance, A549 cell lines resistant to DDP, PTX, and Osi were used. Resistance verification experiments showed that the cell viability of the three resistant cell lines was higher than that of the parent cells under the treatment of the same concentration of drugs, and their IC50 presented a higher value (Figure S1). LINC01605 expression was elevated in drug‐resistant A549 cells (A549/DDP, A549/PTX, A549/Osi) relative to parental cells (Figure 1D). CCK8 assay demonstrated that LINC01605 knockdown decreased cell viability in drug‐resistant cells across various drug concentrations, suggesting that silencing LINC01605 enhanced the sensitivity of drug‐resistant A549 cells to chemotherapeutic agents (Figure 1E–G). Similarly, LINC01605 was upregulated in PTX‐resistant HCC827 cells (Figures 1H and S1), and its knockdown increased the sensitivity of drug‐resistant HCC827 cells to PTX, significantly reducing cell viability at equivalent drug concentrations (Figure 1I).

### 3.2 LINC01605 Is a Diagnostic and Prognostic Biomarker for NSCLC

**FIGURE 2:**
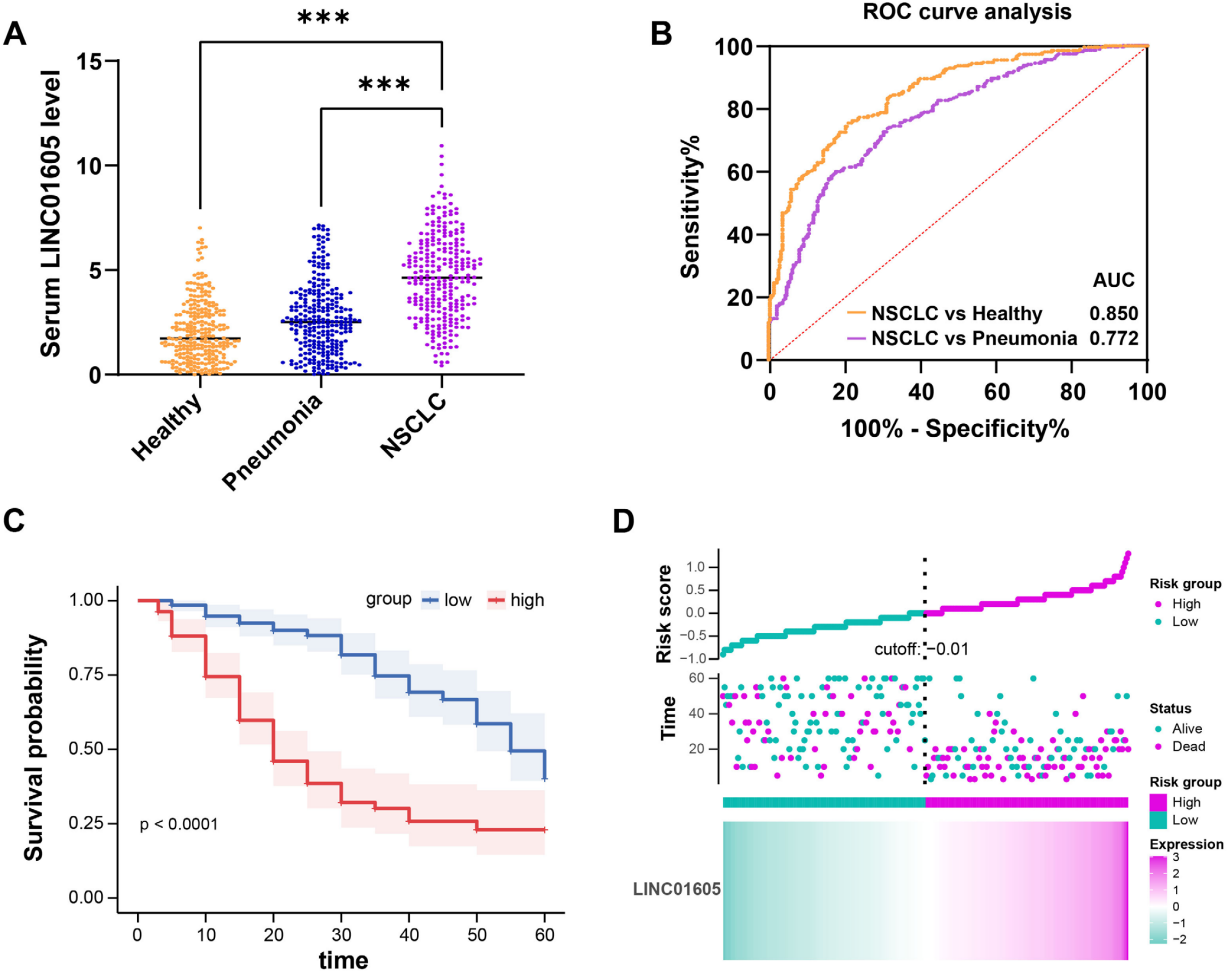
LINC01605 is a Diagnostic and Prognostic Biomarker for NSCLC. (A) The expression of LINC01605 in healthy volunteers, patients with pneumonia or NSCLC. (B) The ROC curve of serum LINC01605 for differentiating healthy individuals from pneumonia and NSCLC patients. (C) Kaplan–Meier(K‐M)curve. (D) Risk analysis of serum LINC01605. ***p < 0.001.

The expression of LINC01605 was detected in healthy volunteers, pneumonia patients, and NSCLC patients, with the highest levels observed in NSCLC patients (Figure 2A). ROC curve analysis revealed that serum LINC01605 had an AUC of 0.850 for distinguishing healthy individuals from NSCLC patients, and an AUC of 0.772 for differentiating pneumonia patients from NSCLC patients (Figure 2B). Kaplan–Meier (K‐M) survival analysis showed a significant decrease in survival rate among NSCLC patients with elevated expression of LINC01605 (Figure 2C). Multivariate Cox regression analysis identified LINC01605 as a risk factor for NSCLC mortality (Table 2). The risk score triplot indicated that high LINC01605 expression was associated with an increased number of deceased patients and elevated risk scores (Figure 2D). These findings collectively establish serum LINC01605 as a prognostic risk marker for NSCLC.

**TABLE 2:** Cox regression analysis.

	HR	Low 95% CI	High 95% CI	*p*
Age	1.053	0.735	1.509	0.777
Gender	1.039	0.729	1.480	0.833
T stage	1.077	0.758	1.530	0.679
N stage	1.083	0.754	1.556	0.667
M stage	1.689	1.131	2.523	0.010
EGFR mutation	1.204	0.836	1.734	0.318
Smoking	1.096	0.768	1.565	0.613
Histological type	1.092	0.763	1.563	0.630
Family history	1.071	0.746	1.538	0.711
LINC01605	1.188	1.094	1.291	< 0.001

Abbreviations: CI, confidence interval; M, distant metastasis; N, lymph node metastasis; T, tumor.

### 3.3 The Upregulation of miR‐7111‐5p Inhibits the Proliferation and Invasion of NSCLC Cells Induced by LINC01605

**FIGURE 3:**
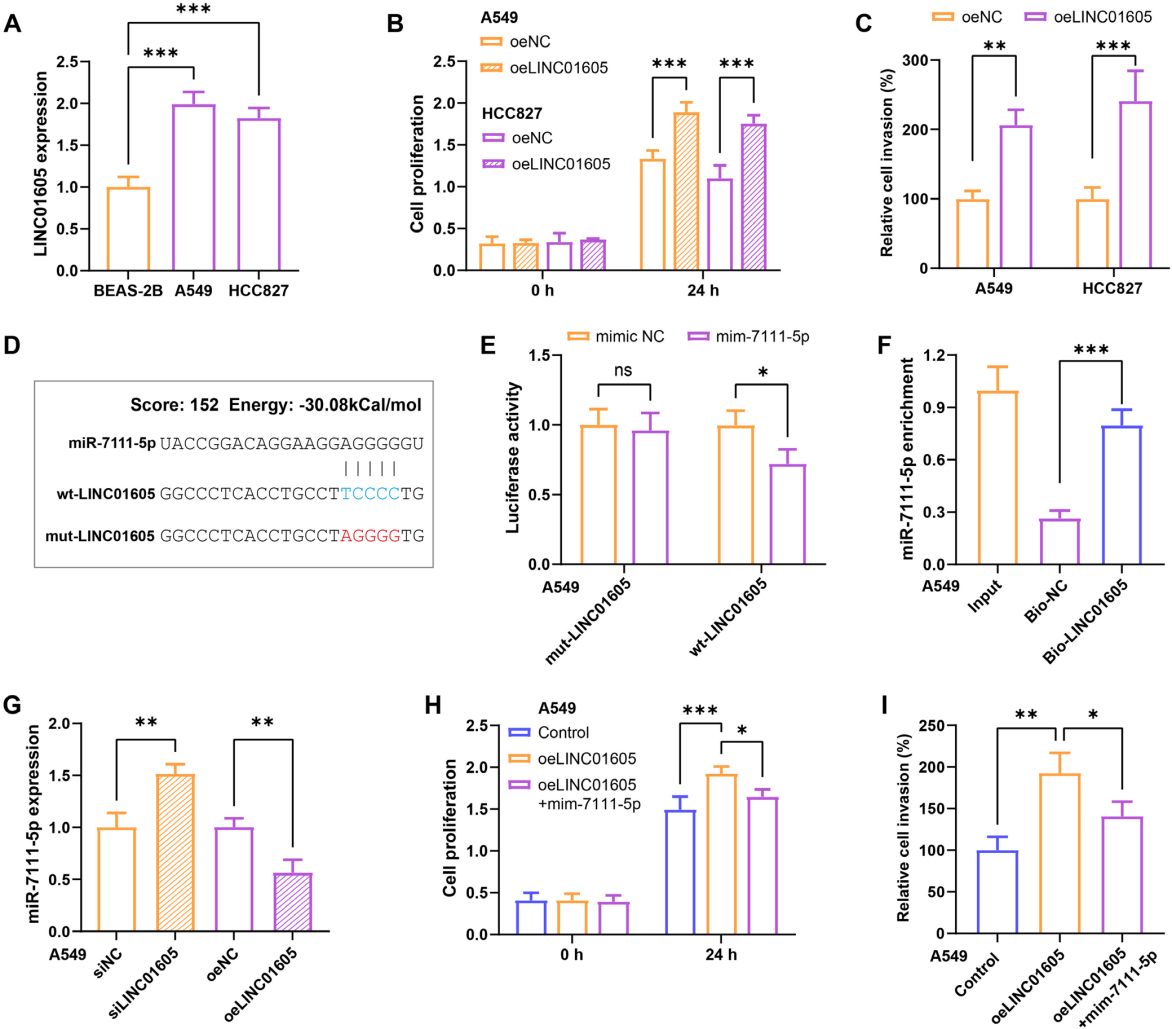
The Upregulation of miR‐7111‐5p Inhibits the Proliferation and Invasion of NSCLC Cells Induced by LINC01605. (A) LINC01605 was highly expressed in NSCLC cells. (B‐C) Overexpression of LINC01605 enhanced the proliferation and invasion of NSCLC cells. (D) Prediction of binding sites of miR‐7111‐5p and LINC01605. (E) Validation of the target relationship between LINC01605 and miR‐7111‐5p via dual luciferase assay. (F) Verification of the interaction between LINC01605 and miR‐7111‐5p via RNA pull‐down experiment. (G) Regulation of miR‐7111‐5p expression by LINC01605. (H–I) The effects of LINC01605 on NSCLC cell functions were reversed by miR‐7111‐5p mimics. *p < 0.05; **p < 0.01; ***p < 0.001; ns, not significant.

Chi‐square testing revealed that high LINC01605 expression was associated with distant metastasis of the tumor (Table 1). LINC01605 expression was markedly upregulated in NSCLC cells (A549 and HCC827) compared to normal lung epithelial cells (BEAS‐2B) (Figure 3A). Following LINC01605 overexpression, the cell reproductive capacity of both A549 and HCC827 cells was significantly increased (Figure 3B), accompanied by enhanced cell invasion ratios in these cells (Figure 3C). These results indicate that LINC01605 further potentiates the proliferative and invasive capacities of NSCLC cells.

Using the lncRNASNP2‐human database, miR‐7111‐5p was predicted as a downstream miRNA of LINC01605 with the lowest binding energy (Figure 3D). Dual‐luciferase reporter assays validated the targeting relationship between LINC01605 and miR‐7111‐5p. Transfection with miR‐7111‐5p mimics significantly suppressed luciferase activity in cells carrying wild‐type LINC01605 constructs, while luciferase activity remained unaffected in cells with mutant LINC01605 constructs (Figure 3E). RNA pull‐down experiments confirmed that miR‐7111‐5p was significantly enriched in biotinylated LINC01605 (Figure 3F). Additionally, LINC01605 overexpression suppressed miR‐7111‐5p expression, while its knockdown elevated miR‐7111‐5p level (Figure 3G), indicating that LINC01605 negatively regulates miR‐7111‐5p expression. Patients with NSCLC were divided into low‐expression and high‐expression groups based on median serum miR‐7111‐5p levels. Chi‐square analysis revealed that low expression of miR‐7111‐5p was associated with larger tumor size, higher incidence of distant metastasis, and elevated EGFR mutation rates (Table 1). Subsequent cell function assays showed that the enhancement of NSCLC cell proliferation and invasion induced by LINC01605 was reversed by co‐transfection with miR‐7111‐5p mimics (Figure 3H,I).

### 3.4 ELK1 Is a Target of the LINC01605/miR‐7111‐5p Axis

Using the miRDB database, genes with a binding score ≥ 90 were identified as potential downstream targets of miR‐7111‐5p. KEGG enrichment analysis revealed their involvement in multiple pathways, with ELK1 showing a strong association with human cancers, including endometrial cancer and hepatocellular carcinoma (Figure S2). Single‐gene enrichment analysis further indicated ELK1's function within the MAPK pathway, regulated by ERK/JNK cascades—both critical in human cancers (Figure S2).

**FIGURE 4:**
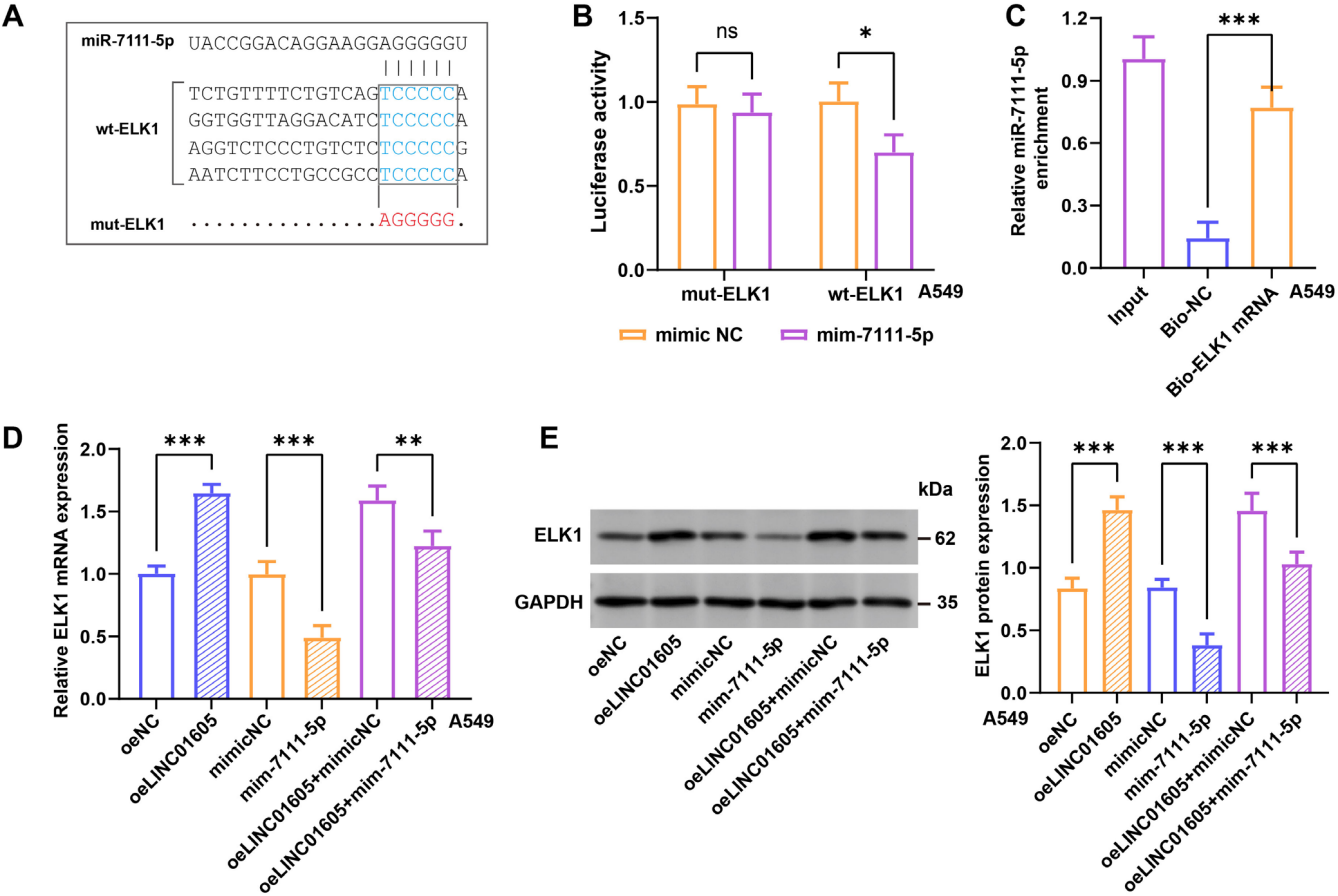
ELK1 is a Target of LINC01605/miR‐7111‐5p. (A) Prediction of binding sites. (B, C) Dual luciferase reporter and RNA pull‐down assays demonstrated that miR‐7111‐5p targeted ELK1. (D, E) The regulation of ELK1 expression by miR‐7111‐5p and LINC01605. *p < 0.05; **p < 0.01; ***p < 0.001; ns, not significant.

We predicted the binding site between miR‐7111‐5p and ELK1 (Figure 4A) and validated their targeting relationship. Transfection with miR‐7111‐5p mimics significantly reduced luciferase activity in cells expressing wild‐type ELK1 but not in those with mutant ELK1 (Figure 4B). RNA pull‐down experiment revealed that miR‐7111‐5p was significantly enriched in biotinylated ELK1 mRNA (Figure 4C). Subsequent RT‐qPCR analysis demonstrated miR‐7111‐5p‐mediated suppression of ELK1 expression. Notably, co‐transfection with miR‐7111‐5p mimics rescued the LINC01605 overexpression‐induced elevation of ELK1 expression (Figure 4D,E).

### 3.5 Silencing ELK1 and Upregulating miR‐7111‐5p Eliminated the Chemotherapy Resistance Induced by LINC01605 in NSCLC

**FIGURE 5:**
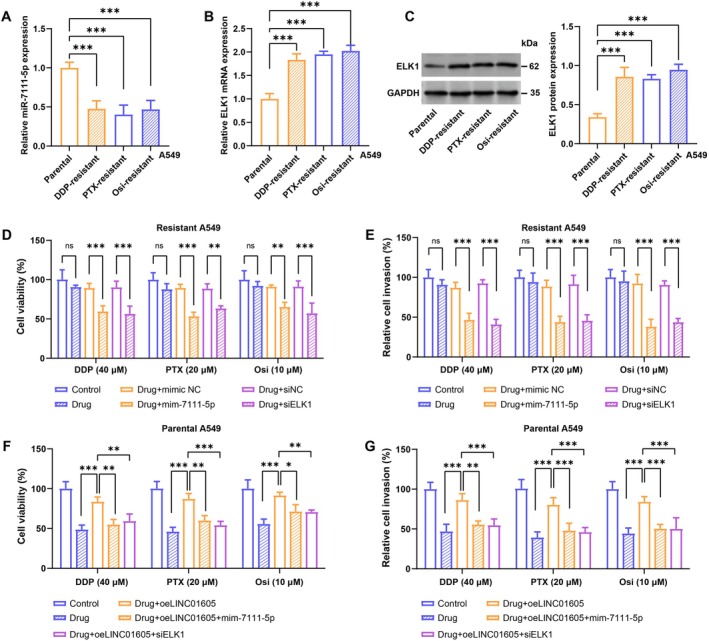
Silencing ELK1 and Upregulating miR‐7111‐5p Eliminated the Chemotherapy Resistance Induced by LINC01605 in NSCLC. (A–C) The expression of miR‐7111‐5p and ELK1 in chemotherapy‐resistant cells. (D, E) Upregulation of miR‐7111‐5p and silencing of ELK1 alleviate chemotherapy resistance in A549 cells. (F, G) Co‐transfection with miR‐7111‐5p mimics or silencing of ELK1 reversed the chemotherapy resistance caused by LINC01605. *p < 0.05; **p < 0.01; ***p < 0.001.

RT‐qPCR and western blotting results revealed decreased miR‐7111‐5p levels (Figure 5A), and increased ELK1 expression in chemoresistant cells (Figure 5B,C). Chemotherapeutic drugs had minimal impact on cell viability and invasion capability in A549 resistant cells, but combined miR‐7111‐5p mimic transfection and ELK1 knockdown significantly sensitized cells to chemotherapy (Figure 5D,E). In normal A549 cells treated with drugs, cell viability and invasion decreased, but this effect was reversed by LINC01605 overexpression. Importantly, this recovery was abrogated by miR‐7111‐5p co‐overexpression and ELK1 silencing (Figure 5F,G).

## 4 Discussion

NSCLC treatment strategies, the most prevalent histopathological subtype of lung cancer, encompass multimodal approaches like surgery, radiotherapy, chemotherapy, immunotherapy, and molecularly targeted therapy, employed either individually or in combination [14]. Surgical resection and radiotherapy are primarily used for localized tumors and early‐stage NSCLC, aiming to physically remove or destroy malignant cells.

For patients ineligible for immunotherapeutic regimens, chemotherapy remains a first‐line treatment option [15], with paclitaxel and cisplatin‐based regimens being standard for advanced NSCLC [16]. The efficacy of cisplatin‐based chemotherapy has also been confirmed [17]. Molecular targeted therapies, such as osimertinib, are effective for NSCLC patients with specific gene mutations [18]. For example, Osi has achieved success in treating EGFR‐mutated NSCLC patients [19]. However, as the duration of drug administration increases, most patients eventually develop drug resistance. Consequently, there is a pressing need for further exploration to identify relevant targets and devise effective therapeutic strategies aimed at overcoming this resistance. In this study, we assessed the expression levels of LINC01605 in the patient's serum and then verified it in vitro. Experiments confirmed that LINC01605 was significantly overexpressed in both chemoresistant NSCLC patients and drug‐resistant A549 cells. Notably, silencing LINC01605 enhanced the sensitivity of drug‐resistant A549 cells to chemotherapeutic agents, suggesting that LINC01605 serves as a risk factor contributing to chemotherapy resistance in NSCLC.

LncRNAs and miRNAs, two principal members of the non‐coding RNA family, have been shown to play crucial roles in tumorigenesis and the progression of NSCLC. Previous research has identified LINC01605 as a tumor‐promoting long non‐coding RNA in colorectal cancer [20]. Similarly, in breast cancer models, stable knockdown of LINC01605 has been demonstrated to significantly inhibit malignant phenotypes, including cell proliferation and invasive potential [13]. However, its role in NSCLC had remained unreported until now. Here, we demonstrate that LINC01605 is highly expressed in NSCLC patients, and this elevated expression is associated with a poor prognosis. Given that tumor metastasis poses a major challenge in NSCLC treatment, we further examined its functional impact. Our results reveal that overexpression of LINC01605 enhances both the proliferative and invasive capacities of NSCLC cells, suggesting its potential involvement in promoting aggressive tumor behavior.

Similarly, miR‐10a overexpression has been shown to drive the malignant progression of NSCLC cells in vitro [21]. Furthermore, Yin et al. demonstrated that miRNA‐221 promotes NSCLC growth and invasion by suppressing TIMP2 expression [22]. In order to clarify the regulatory mechanism of LINC01605 in chemotherapy resistance in NSCLC, we identified the LINC01605/miR‐7111‐5p/ELK1 axis through bioinformatics analysis. Notably, studies have revealed that ELK1 is frequently overexpressed in various cancers and acts as an oncogene, enhancing cancer cell proliferation, invasion, and survival [23]. ELK1 expression is significantly elevated in breast cancer tissues and is implicated in chemoresistance across different cancer types [24]. Research indicates that ELK1 is upregulated in PDX‐R and PC9G cells, and its inhibition restores sensitivity to gefitinib in drug‐resistant cells [25]. In this study, we confirmed that LINC01605 suppresses miR‐7111‐5p expression while upregulating ELK1. Chemoresistant cells exhibited significant downregulation of miR‐7111‐5p and concurrent upregulation of ELK1. Importantly, either miR‐7111‐5p overexpression or ELK1 knockdown reversed the chemoresistance induced by LINC01605, offering new insights into strategies for overcoming chemoresistance in NSCLC. However, the limitations of this study must be acknowledged. The reliance on a single drug‐resistant A549 cell line may not fully capture the heterogeneity of NSCLC. Future studies should incorporate multiple NSCLC cell lines representing different histological subtypes to enhance the generalizability of our findings. Nevertheless, our study provides compelling evidence that LINC01605 is involved in chemotherapy resistance in NSCLC and elucidates a novel ceRNA mechanism centered on the miR‐7111‐5p/ELK1 axis.

Additionally, we observed that inhibiting the LINC01605/miR‐7111‐5p/ELK1 axis did not fully restore drug sensitivity, strongly suggesting the involvement of compensatory resistance mechanisms. These may include the activation of parallel survival pathways (e.g., PI3K/AKT or JAK/STAT signaling) [26, 27, 28], enhanced drug efflux mediated by ABC transporters [29], or the persistent existence of drug‐resistant cancer stem cell subpopulations [30, 31]. Moreover, factors from the tumor microenvironment, such as fibroblast‐mediated protection or immune evasion [32, 33], may also play significant roles that remain unaddressed in our cell‐autonomous model. Therefore, drug resistance in NSCLC appears to be jointly regulated by a complex, multifactorial network, with ELK1 representing just one of several key nodes. In subsequent studies, we plan to perform transcriptomic analysis (RNA‐seq) on cells following intervention of the LINC01605/miR‐7111‐5p/ELK1 axis to systematically identify alternative signaling pathways that are activated.

## 5 Conclusions

In conclusion, our study demonstrates that LINC01605 is highly expressed in chemotherapy‐resistant NSCLC patients. Mechanistically, LINC01605 promotes chemoresistance in NSCLC cells by regulating the miR‐7111‐5p/ELK1 axis. These findings suggest that LINC01605 holds potential as a biomarker for predicting chemoresistance in NSCLC.

## Conflicts of Interest

The authors declare no conflicts of interest.

## Supporting information


**Figure S1:** (A–C) Verification of drug‐resistant A549 cells. (D) Verification of drug‐resistant HCC827 cells.

**Figure S2:** (A, B) KEGG enrichment analysis of multiple genes or a single gene.

## Data Availability

The data that support the findings of this study are available from the corresponding author upon reasonable request.
