# Supplementary figures and images for: LINC01605 Predicts the Poor Prognosis of Non‐Small Cell Lung Cancer and Promotes Chemotherapy Resistance by Regulating miR‐7111‐5p/ELK1
Source: Kaohsiung J Med Sci. 2026 May 15:e70224. Online ahead of print. doi: 10.1002/kjm2.70224 (PMC13399733; doi:10.1002/kjm2.70224)

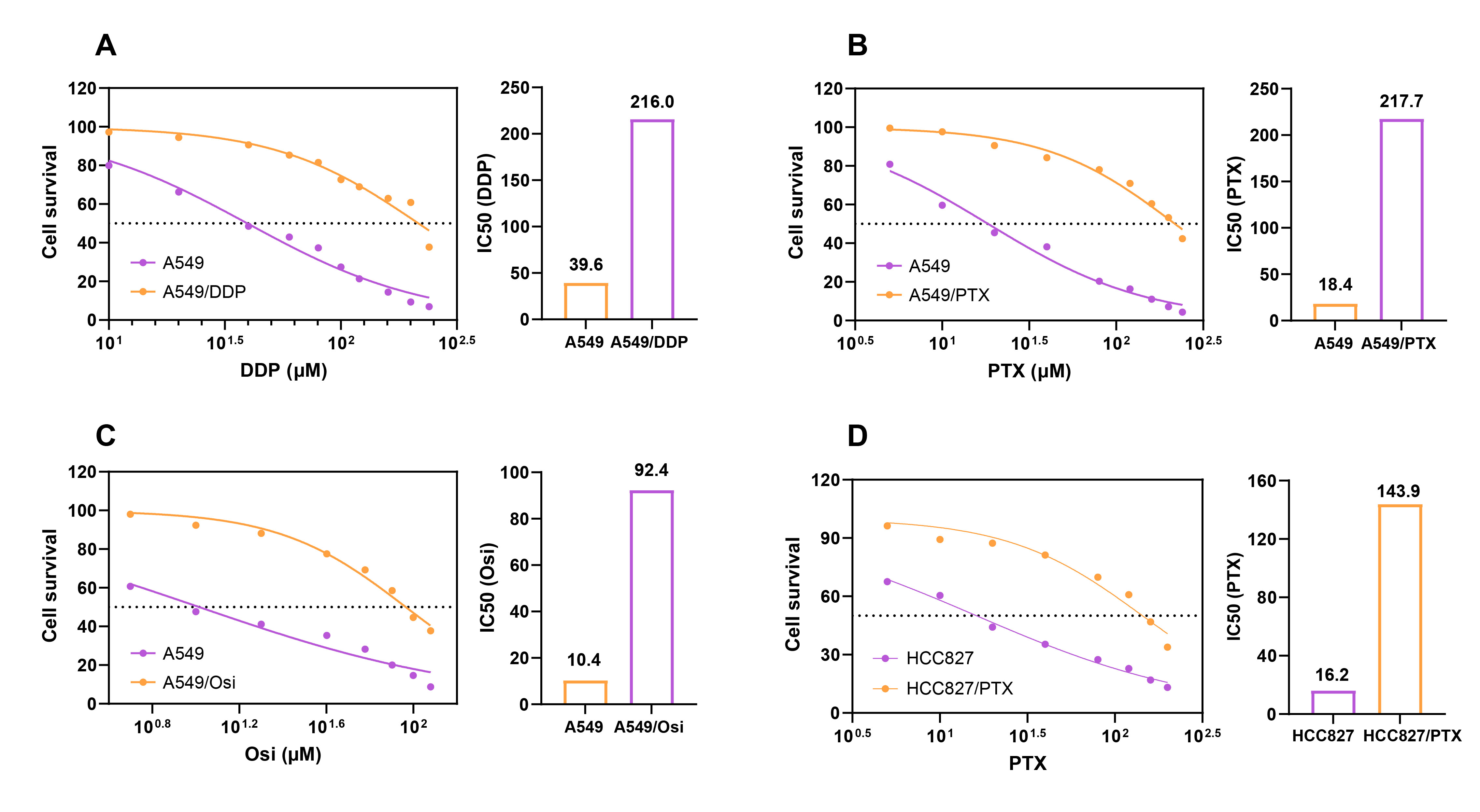

Supplement: Supplementary file 1 — Figure S1: (A–C) Verification of drug‐resistant A549 cells. (D) Verification of drug‐resistant HCC827 cells. [file KJM2-9999-e70224-s002.jpg]

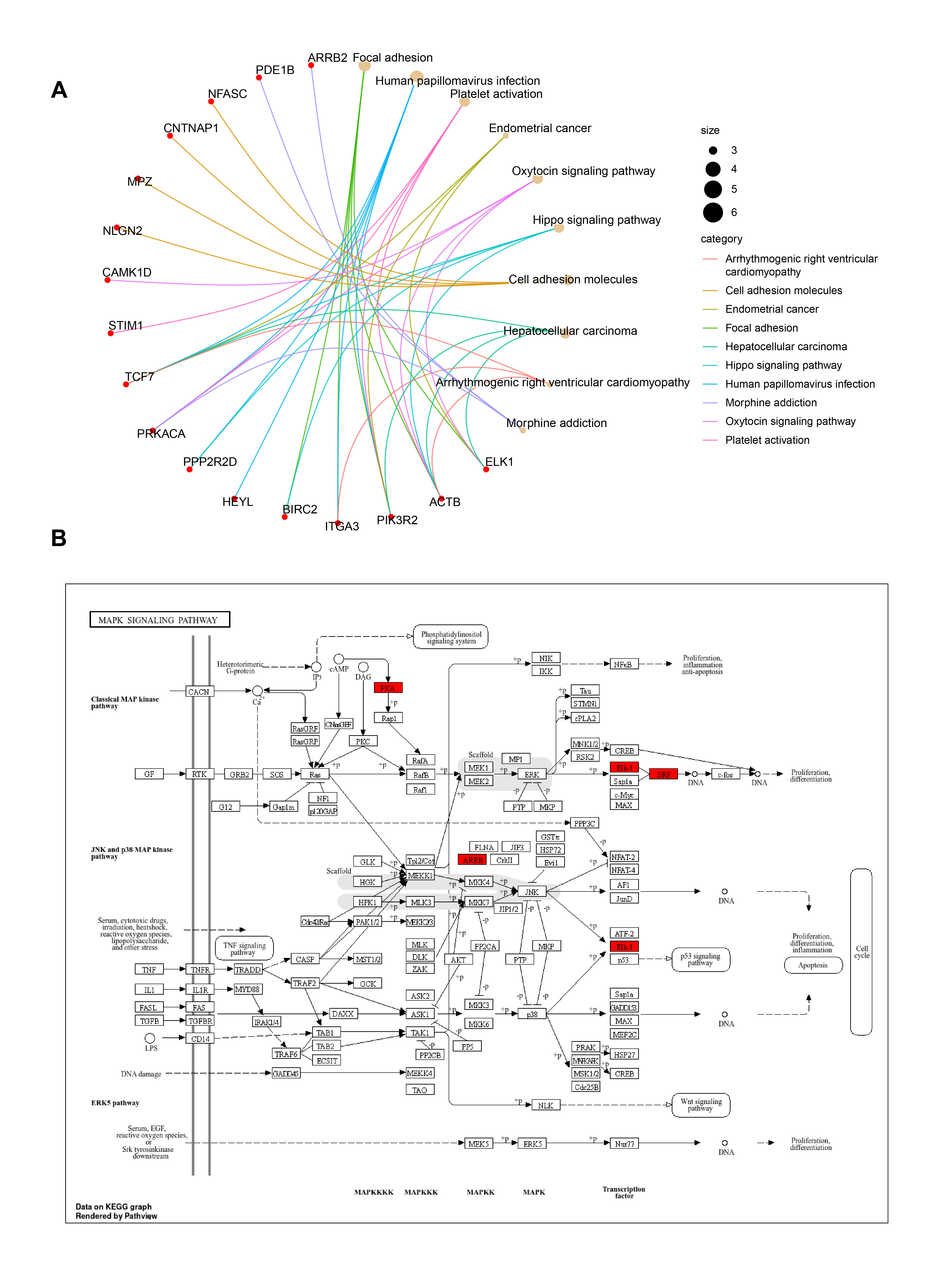

Supplement: Supplementary file 2 — Figure S2: (A, B) KEGG enrichment analysis of multiple genes or a single gene. [file KJM2-9999-e70224-s001.jpg]
